# Ectopic pregnancy and epithelial to mesenchymal transition: is there a link?

**DOI:** 10.1530/REP-20-0542

**Published:** 2020-11-30

**Authors:** Heather Flanagan, Chih-Jen Lin, Lisa L Campbell, Paddy Horner, Andrew W Horne, Norah Spears

**Affiliations:** 1 Biomedical Sciences, University of Edinburgh, Edinburgh, Scotland; 2 MRC Centre for Reproductive Health, University of Edinburgh, Edinburgh, Scotland; 3 Population Health Sciences, University of Bristol, Bristol, UK; 4 NIHR Health Protection Research Unit in Behavioural Science and Evaluation, University of Bristol, Bristol, UK

## Abstract

Ectopic pregnancy (EP) is defined as the implantation of an embryo outside of the uterus and is a leading cause of first trimester maternal mortality and morbidity. This article discusses a possible role for epithelial to mesenchymal transition in the pathogenesis of EP, given the notable similarity of protein expression between the two processes.

## Introduction

Ectopic pregnancy (EP) is defined as the implantation of an embryo at a location outside of the uterine cavity (Sivalingam *et al.* 2011). EP occurs in 1–2% of all pregnancies and is a leading cause of first-trimester death worldwide. The most common implantation site for an EP is the Fallopian tube, commonly referred to as a tubal ectopic pregnancy (tEP) (Sivalingam *et al.* 2011). Multiple risk factors predispose to tEP, such as smoking, but a unifying mechanism has not yet been identified. Current research on tEP suggests that increased embryo-receptivity is secondary to an abnormal tubal microenvironment, but there are still gaps in the literature and more research is necessary to understand the processes involved in the development of a tEP.

Epithelial to mesenchymal transition (EMT) is a biological process whereby epithelial cells undergo loss of polarity and cell–cell adhesion, then adopt mesenchymal characteristics such as migration, invasion and resistance to apoptosis (Bilyk *et al.* 2017). EMT is associated with normal functions such as tissue regeneration and embryogenesis but the full role of EMT in various organs are not yet fully understood. Endometrial EMT is required for embryo implantation in normal intrauterine pregnancy (Bilyk *et al.* 2017). EMT has also been associated with disease states in the reproductive tract such as ovarian cancer and adenomyosis (Bilyk *et al.* 2017).

**Figure 1:**
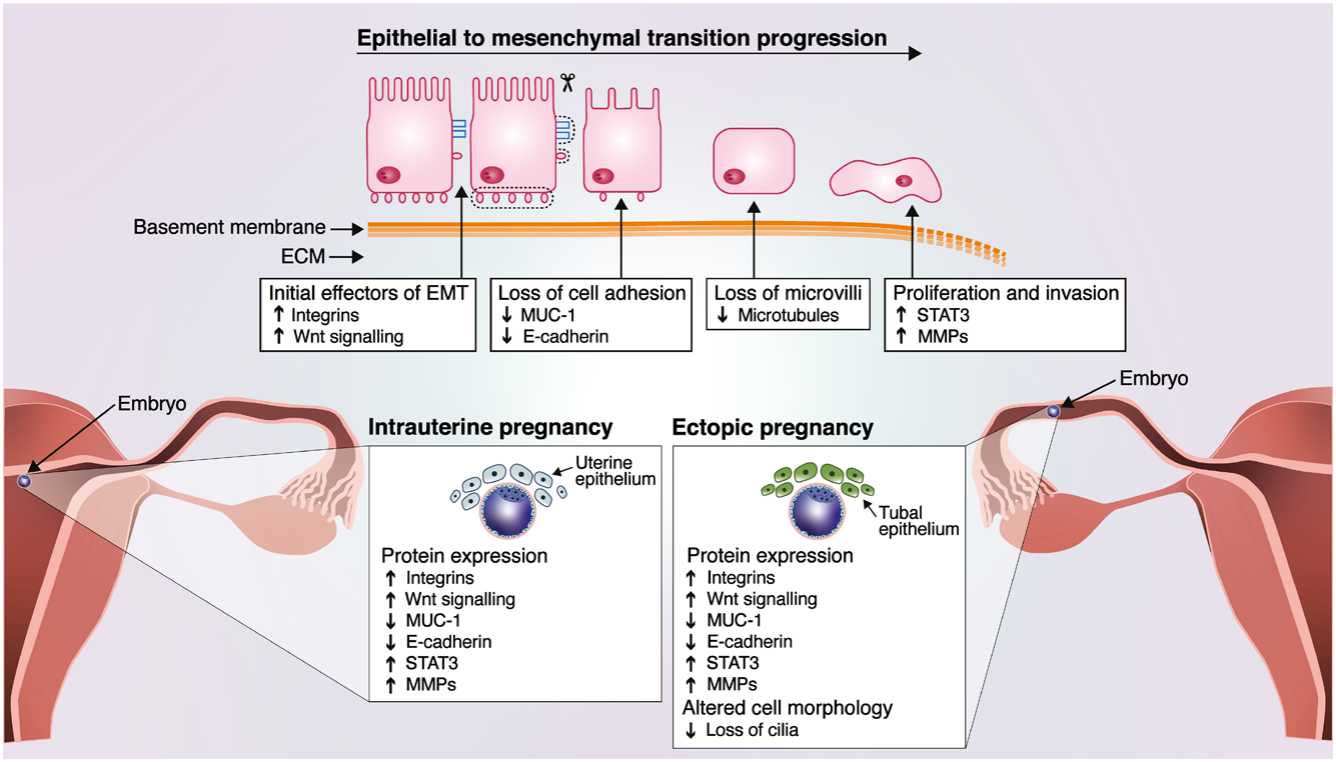
Similarities between EMT, EP and intrauterine pregnancy. Epithelial cells displaying polarity are held together by adhesion proteins and tight junctions. Induction of EMT effector proteins such as integrins and Wnt signalling leads to a downstream loss of cell adhesion proteins; blue and pink receptors represent MUC-1 and E-cadherin receptors, respectively: scissors represent a loss of these proteins. Due to the loss of cell adhesion and a loss of microvilli, epithelial cells resort to a mesenchymal-like state. This leads to the upregulation of STAT3 and MMPs which results in basement membrane degradation and induces proliferation and invasion of mesenchymal cells which are loosely arranged in the extracellular matrix (ECM). Both EP and intrauterine pregnancies have similar protein expression patterns to those expressed in the EMT process.

Here, we put forward the hypothesis that tEP occurs as a result of EMT in the epithelial cells lining the Fallopian tube, with these changes enabling ectopic embryo implantation (Fig. 1).

## Link between tEP and EMT

There are many similarities in protein expression between EMT and tEP including β-integrins, Wnt, Mucin-1 (MUC-1), E-cadherin, signal transducer and activator of transcription (STAT3), and matrix metalloproteases (MMPs) (Shaw *et al.* 2010, Bilyk *et al.* 2017). Initial induction of EMT is under the control of several factors. The upregulation of β1-integrin through the TGF-β pathway is essential for the onset of EMT (Yeh *et al.* 2010). Wnt signalling is also described as an initial inducer of EMT, releasing β-catenin from E-cadherin which acts as a downstream factor increasing the transcription of EMT-related proteins (Bilyk *et al.* 2017). Cell adhesion proteins play a significant role in EMT, with a loss of cell-adhesion caused by decreased expression of MUC-1 and E-cadherin (Guaita *et al.* 2002, Bilyk *et al.* 2017). As EMT progresses, the basement membrane degrades, while proliferation and invasion are promoted. These events are facilitated by the upregulation of matrix metalloproteases (MMPs) and STAT3, both of which promote proliferation and invasion during EMT (Bilyk *et al.* 2017).

Similar to the process of EMT, β1-integrin is upregulated in the endometrial luminal epithelium during implantation in intrauterine pregnancy and has also been noted to be increased in the cytoplasm of Fallopian tube epithelial cells in tEP (Shaw *et al.* 2010, Jiang *et al.* 2019). Wnt plays an essential role in embryo attachment in endometrial luminal epithelial cells and Wnt upregulation has been shown to increase trophoblast attachment in Fallopian tubal epithelial cells* in vitro* (Kodithuwakku *et al.* 2012). Proteins affecting cell adhesion have a major role in tEP (Shaw *et al.* 2010). In the endometrial luminal epithelium, the ‘anti-adhesive’ action of MUC1 is downregulated during implantation, enabling embryo adhesion: likewise, in tEP, there is a marked reduction in MUC-1 expression in epithelial cells of the Fallopian tube, which again could facilitate attachment of embryos due to its ‘anti-adhesive’ action (Shaw *et al.* 2010). There is also a loss of E-cadherin in tEPs (Shaw *et al.* 2010, Jiang *et al.* 2019). MMPs play an important role in normal implantation in the extracellular matrix of the uterine luminal epithelium and tEP has been shown to have a similar MMPs expression milieu in the epithelial and smooth muscle cells of the Fallopian tube at the site of implantation (Qiu *et al.* 2011). Furthermore, the STAT3 pathway, expressed in the uterine luminal epithelial cells during normal implantation, can be activated by leukaemia inhibitory factor, LIF (Krishnan *et al.* 2013). LIF exposure increases trophoblast spheroid adhesion in Fallopian tube cells* in vitro* (Krishnan *et al.* 2013): LIF also promotes EMT by activating the STAT3 pathway (Yue *et al.* 2016). Not all EMT protein expression changes are mirrored by alterations during tEP. The SLIT/ROBO genes, described as tumour suppressor genes, are involved in the EMT process (Duncan *et al.* 2010), and endometrial SLIT/ROBO expression has been noted to change in a temporal and spatial manner across the menstrual cycle, but the examination of Fallopian tube epithelial cells, has found no difference in expression of SLIT/ROBO between samples with and without tEP (Duncan *et al.* 2010). Other markers involved in the EMT process, such as Slug, Snail, and Twist, are expressed in the endometrium but have not yet been analysed in the Fallopian tube epithelium or tEP. Although, the link between tEP and EMT is marked, it is non-the-less possible that EMT is a consequence rather than a cause of the presence of tEP, a phenomenon that would also be of interest.

## Fallopian tube secretory cell expansion and EMT

The Fallopian tube epithelium is comprised of two different cell types, secretory cells and ciliated cells. Fallopian tube secretory cells are the cells of origin for ovarian carcinoma, the most common cancer in the female pelvic organs: these secretory cells have unique biology, co-expressing both epithelial and mesenchymal markers (Yamamoto *et al.* 2016). Secretory cell outgrowth (SCOUT) refers to secretory cell expansion of at least 30 cells of secretory epithelial type, creating a lesion of only secretory cells compared to a mosaic of ciliated and secretory cells in normal Fallopian tubes (Yamamoto *et al.* 2016). It is widely accepted that the origin of EMT for ovarian carcinoma is derived from the Fallopian tube secretory cells, suggesting that an altered Fallopian tube cellular structure and environment could be present years before an ovarian cancer diagnosis. Furthermore, there have been reports of the risk of ovarian carcinoma being increased in women with a history of tEP, although this is more controversial, with other literature failing to find such a link (Stewart *et al.* 2020).

Although, SCOUT’s have been researched in ovarian carcinoma; the pathophysiology and implications on tubal factor fertility of SCOUT’s have not yet been determined. SCOUTS have been recently demonstrated to have upregulated LEF1 (Schmoeckel *et al.* 2017). LEF1 activates the transcription of hallmark EMT effectors including N-cadherin, Vimentin, and Snail (Santiago *et al*. 2017). SCOUTS can be also found throughout the Fallopian tube (Fig. 1A in Schmoeckel *et al.* 2017). Yamamoto *et al.* (2016) describe an altered tubal epithelial cell microenvironment associated with immortalisation in SCOUTs and suggest that these aberrations in gene expression may even occur prior to and in the absence of serous carcinoma (Yamamoto *et al.* 2016). When secretory cells become neoplastic, they can exhibit EMT characteristics such as increased proliferation, a loss of nuclear polarity and the ability to migrate (Yamamoto *et al.* 2016). Additionally, tEP is associated with a loss of ciliation, and so embryo implantation in tEP cases may be proceeding at sites where that cilia loss has occurred (Shaw *et al.* 2010, Yamamoto *et al.* 2016). Therefore, it could be hypothesised that EMT occurring at the site of SCOUTs may lead to an ectopic implantation in the Fallopian tube.

## Smoking and tEP

Cigarette smoking increases the risk of tEP to 1.7–3.9% of pregnancies in smokers (Sivalingam *et al.* 2011). In line with our hypothesis, cigarette smoke is widely associated with EMT, including having been shown to induce EMT in the reproductive system. Nicotine increases EMT in human ovarian carcinomas (Jeon *et al.* 2016). Cotinine is an active metabolite of nicotine. Exposure of a Fallopian tube epithelial cell line to cotinine causes decreased Bcl2-associated agonist of cell death (BAD) expression, leading to decreased Bcl2 expression; reduced BAD expression is also implicated in EMT (Horne *et al.* 2014). Another cigarette smoke component, Benzo(a)Pyrene (BaP), is known to inhibit endometrial cell apoptosis leading to impaired endometrial function. It has not been investigated if this impaired function is secondary to an EMT process but given the ability of BaP to induce EMT in colonic epithelium by altering the Wnt/β-catenin pathway: it would seem possible (Yi *et al.* 2019). To date, the effects of BaP exposure on the Fallopian tube epithelium have not yet been researched. We hypothesise that the components of cigarette smoke may induce EMT in Fallopian tube epithelium, explaining the increased risk of tEP associated with cigarette smoking.

## Conclusion

In summary, EMT is likely a common process in the Fallopian tube, which could explain the pathophysiology of EP, providing a unifying a mechanism behind multiple risk factors for tEP. There are similarities in protein expression between EMT and tEP, including integrins, Wnt, MUC-1, E-cadherin, STAT3 and MMPs. There is also evidence to suggest EMT occurs in the secretory cell outgrowths of the Fallopian tube causing progression to ovarian carcinoma, although the implications on fertility and reproductive consequences in relation to an ectopic pregnancy are largely unknown. Finally, there is a significant link between cigarette smoke components and EMT in the female reproductive system and we hypothesise this may extend to the Fallopian tube epithelium. In conclusion, the similarities in the literature between EMT and tEP suggests that further research is required to understand if there is a clear link between tEP and EMT, and if so, whether EMT is a causative factor in tEP.

## Declaration of interest

The authors declare that there is no conflict of interest that could be perceived as prejudicing the impartiality of this article.

## Funding

This work was supported by a UK Medical Research Council http://dx.doi.org/10.13039/501100000265 Centre Grant (to A H, N S, C J L, L C; MR/N022556/1, 2018-22) and a joint Medical Research Council http://dx.doi.org/10.13039/501100000265 /Ectopic Pregnancy Trust PhD Fellowship to H F.

## Author contribution statement

H F conceived and drafted the manuscript; C J L, L L C and P H helped draft the manuscript; A W H and N S conceived and helped draft the manuscript. All authors read and approved the final version of the manuscript. A W Horne and N Spears contributed equally to this work.
